# Assessing trait emotional intelligence and its relationship with stress and health behaviour in the education sector: An empirical study from Uttarakhand, India

**DOI:** 10.12688/f1000research.131306.4

**Published:** 2023-11-08

**Authors:** Mamta Pandey, Deepti Sharma, NK Kamboj

**Affiliations:** 1 Faculty of Management, Uttaranchal University, Dehradun, Uttarakhand, 248001, India; 2 School of Physical Sciences, DIT University, Dehradun, Uttarakhand, 248001, India

**Keywords:** Emotional intelligence, Trait EI, Ability EI, stress, health behaviour, teachers, education sector, mental health

## Abstract

Background: Emotional intelligence of teachers can affect their mental and physical health as well their performance in school. Both emotional intelligence and health behavior can have an impact on stress. The majority of Indian studies have examined only one type of teachers, have used indigenous scales which are not internationally valid, and have not studied health behaviour. The role of age and gender on emotional intelligence is also a debatable subject which requires larger studies

The present study was undertaken to evaluate the trait emotional intelligence, stress and health behaviour of teachers and to determine their inter-relationship and to assess the role of demographic and professional attributes on emotional intelligence.

Methods: Teachers from different schools, colleges and professional institutes situated in Dehradun and nearby towns in the state of Uttarakhand, India were evaluated by internationally valid tools for the three parameters.

Results: Emotional Intelligence of teachers has no relation with age, gender, educational qualification, level of teaching or type of institute. It has a negative correlation with stress and a positive correlation with health behaviour. Further, health behaviour is inversely related to stress.

Conclusions: Assessment of emotional intelligence and health behaviour of teachers should be a part of their routine evaluation and training so that specific interventions to reduce stress and to improve their overall health and performance can be appropriately planned.

## Introduction

Education is not the filling of a pot, but the lighting of a fire.

- WB Yeats.

Right from the first introduction to alphabet, to the discussion on the origin of universe, teachers open the minds of the students and lead them on to a journey of unravelling the mysteries of not only the outer world, but their inner self. But how well do we actually know those who impact almost all aspects of our life? A lot of work has been done on the professionalism of teachers, how well are they trained, their educational qualifications, their skill for imparting theoretical and practical knowledge, etc. And all of it is absolutely necessary. But equally important, if not more, is a study into their personal well - being, their thoughts and emotions and feelings, their weaknesses, their vulnerabilities, and their health. For, only a teacher who is happy and healthy can become a role model for their students. Assessment of emotional intelligence, stress and health behaviour of teachers and their inter-relationship forms the basis of this study.

Emotional intelligence (EI) is self-perception about one’s own emotions or the ability to understand and regulate one’s own and others’ emotions. Since the publication of the seminal works of Mayer and Salovey, and Reuven Bar-On, and publication of Daniel Goleman’s book on this topic in the 90s, the field has seen rapid development of the conceptual framework, theoretical models, and measuring tools. These three researchers have proposed the three main models and definitions of this construct and the measurement tools based on these models have been widely used by researchers across the world. While Mayer and Salovey define EI as an ability to perceive and regulate one’s own and others’ emotions, ^1^ Bar-On defines it as the composite of competencies and skills which help us in dealing with social and environmental pressures. ^2^ According to the Goleman model, EI is a competency to manage emotions for motivation which contributes to effective performance at work. ^3^ Petrides and colleagues proposed a new ‘trait model’ which defines EI as self -perception of emotions and behaviour in emotional situations and suggested it as a part of personality. ^4^ A large body of research focusing on its association and correlation with other behaviour aspects, personality traits, health indices and organizational skills ^5^ has established its scientific credentials and generated interest from academics, practicing psychologists, media, corporate world and public at large. Dana Ackley, in a recent review has given a succinct and lucid introduction to the concept of emotional intelligence and its various theories, models and some practical applications. ^6^ In simplest terms, ‘Emotional Intelligence’ is the intelligent use of emotions. O’Connor *et al.,* ^7^ have summarized the various tests for measuring EI and their relative strengths and weaknesses, and their appropriate uses for both academic and professional purposes. The classification of the construct into ability, trait and mixed models is now standard and is based on the type of tool used for measurement. The trait EI model conceptualizes it as a personality trait which is distinct from cognitive intelligence and abilities. ^8^ Petrides *et al.* ^5^ found stronger association of trait EI and human behavioural patterns as compared to ability EI. Dolev and Lesham ^9^ showed that training programmes are effective in improving EI which improves teachers’ performance, their sense of meaningfulness and their relations with students.

A lot of research has shown that women have better emotional intelligence than men, and age has a positive correlation with EI, but this is not universally proven and conflicting results regarding the role of gender and age leave this issue wide open for discussion. ^10^^–^^14^ Extremera, Fernández-Berrocal and Salovey reported higher ability EI in women than men. They noted that studies employing self-report measures either do not find this difference or even sometimes report men as having higher scores than women. ^15^ There is inadequate explanation for some of the associations like general intelligence, professional qualifications, or level of teaching with EI. As there is poor correlation between cognitive intelligence and trait EI, it may be plausible that teachers at different levels of institutes, although differing in general intelligence, may have similar EI, but it needs empirical proof from larger studies.

It has been well established that individuals with high level of emotional intelligence experience less stress. ^16^^,^^17^ Several studies, both from India and abroad, done among teachers have shown a negative correlation between EI and stress. ^18^^,^^19^ Very few Indian studies, however, have taken participants from more than one type or level of educational institutes which makes it difficult to generalize their results. ^20^

Vickers *et al.*, proposed a multidimensional model of health behaviour. ^21^ It comprised of preventive health behaviours including two specific dimensions of wellness maintenance behaviours and accident control behaviours and risk-taking behaviours with two specific dimensions of traffic-related risk taking and use of potentially harmful substances. Connor defined health behaviours as activities undertaken for the purpose of preventing or detecting disease or for improving health and wellbeing. ^22^ Some of the examples include smoking, alcohol use, diet, physical activity, sexual behaviours, physician visits, medication adherence, screening and vaccination. Studies done on students have shown a positive linkage of EI, coping and health behaviours. ^23^ Gilbert *et al.*, in a study from France, compared teacher’s health/risk behaviours to those of non-teachers and found that teachers’ health behaviour was better than other professionals. ^24^ Espinosa & Kadić-Maglajlić, in a structural equation model, showed an inverse releationship between EI and unhealthy behaviours. ^25^ Gillan *et al.*, showed that teachers with healthy food habits chose more task-oriented coping and regular physical activity was associated with less perceived stress and more effective coping. ^26^ Some studies in the developed countries have also utilized teachers as a vehicle to improve overall school health and designed national programs accordingly to target a broader audience of students. ^27^ Sorensen *et al.*, demonstrated a positive effect of a school-based intervention designed to promote tobacco control among teachers in the Indian state of Bihar. ^28^ While previous research has shown a positive correlation of EI with positive health behaviour, and a negative correlation with negative health behaviours, no such study has been conducted among teachers in India.

## Objectives

1. To measure Trait Emotional Intelligence of teachers in different educational institutes by Schutte Self-reported Emotional Intelligence Test (SSEIT).
2. To measure Stress among teachers by Perceived Stress Scale (PSS).
3. To assess Health Behaviour of teachers by Health Behaviour Checklist (HBC).
4. To determine correlation of Trait Emotional Intelligence with Stress and Health Behaviour.
5. To evaluate the effects of parameters like age, gender, educational qualifications, and level of teaching institute on EI.

## Hypothesis and research model

Based on the conceptual framework from review of literature and objectives of the study, following hypothesis have been formulated:

H1. There is no significant difference in EI based on age, gender, professional qualification, and level of teaching. As the construct of trait EI is considered separate from cognitive intelligence, it should not be affected by educational qualification and other professional attributes of teachers.
H2. There is an inverse correlation between EI and perceived stress.
H3. There is a positive correlation between EI and health behavior, and negative correlation between stress and health behavior.

**Figure 1.**
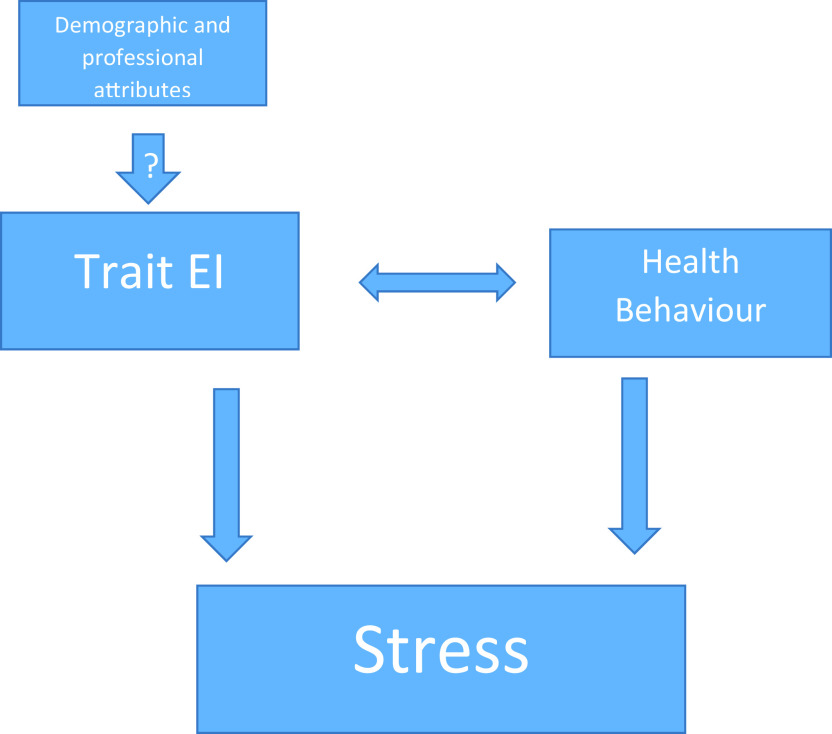
Research Model.

The proposed work can be summarized with a diagrammatic model depicting the relationship between the parameters ( Figure 1). The effect of demographic and professional attributes on trait EI is questionable and our hypothesis suggests they don’t impact trait EI. Further, trait EI and health behavior are two independent variables, which positively impact each other and have a negative correlation with stress.

## Methods

### Study design

This was a questionnaire based cross sectional empirical study to assess the three parameters among teachers and to determine their inter-relationship. The study enrolled teachers from different teaching institutes in and around the town of Dehradun in the north Indian state of Uttarakhand by both online and offline route. The study was conceptualized in January 2022 with the background of Covid induced lockdown. Data collection by online route began in February 2022 and after the resolution of Covid wave, offline collection was started. Data collection was completed by July 2022.

### Participants

The sample population consisted of teachers of both genders, of different educational institutes including primary schools, senior secondary schools, colleges and professional institutes of Dehradun and neighbouring areas in the northern state of Uttarakhand, India. All individuals above 18 years of age who were teaching in any type of educational institute were included, non -teaching staff and trainee teachers were excluded. The teacher population in the town was estimated to be around ten thousand by various media sources in the public domain and a minimum sample size of 500 (5% of population) was planned.

### Data collection

Participants were contacted and requested to fill the questionnaires by both online and offline route. A copy of the questionnaire can be found under *Extended data*. ^31^ For the online questionnaire, a google form was created asking demographic profiles and including all three scales. For the online survey, participants were identified initially among the authors’ friends and family members, colleagues, previous and current educational institutes and subsequently through various social media platforms. Participants were sent the link to Google forms by phone (Whatsapp) and were required to sign in with mail id. For proper representation and randomization, a list of 48 different schools and institutes in and around the town of Dehradun was made with 24 institutes each in government and private sector comprising of 6 institutes each in the 4 predefined levels of teaching. From each institute, 5 male and 5 female teachers were randomly selected and physically contacted and were given printed questionnaires so that adequate number of participants from both genders and from different level of institutes in both government and private sector could take part in the study. All participants were informed in detail about the study objectives and all data were collected confidentially. Consent was taken from each participant with explicit information that the data will be used for the sole purpose of the present research and any publication related to it.

Following standard tools were used for the study.

1) For measuring trait EI, Schutte Self-report Emotional Intelligence Test (SSEIT) was used. ^29^ This scale measures 4 facets of emotional intelligence as defined by Mayer and Salovey. It uses a 5-point Likert scale ranging from 1 (strongly-disagree) to 5 (strongly-agree) and comprises of 33 questions. Although some later researchers have argued for using these components as a four -factor analysis of this tool, Schutte *et al.* themselves have advocated use of the composite scale as single factor for scoring EI.
2) For measuring Stress – Perceived Stress Scale (PSS) – by Cohen *et al.* was used. ^30^ It comprises of 14 questions, each with 5 possible answers in 5-point Likert scale.
3) For measuring health behaviour, health behaviour checklist by Vickers was used. ^21^ This tool has 40 questions with answers on a 5-point Likert scale ranging from 1(disagree strongly) to 5 (agree strongly). A few questions were reframed given the widespread use of mobile phones nowadays (in place of fixed landlines) and internet and to suit the weather conditions in India (written as extremes of temperature in place of ‘chilled’).

### Data analysis

For testing reliability of scales, Cronbach alpha was calculated. For determining correlation between the three parameters individually as well as the relationship between EI and the continuous variable age, Pearson’s coefficient ‘r’ was calculated. For determining the effect of gender and type of institute on EI, t-test was employed to find the significance of difference between the two groups. For determining the effect of educational qualification and level of teaching, ANOVA test was used to find any significant difference among the four groups. Finally, a multiple regression analysis was carried out to assess the relationship between the independent variables EI and health behaviour and the dependent variable stress.

Ethical considerations

Ethical approval for the study was obtained from Uttaranchal University Research Ethics Board (No- UU/DRI/REB/2023/004). Written informed consent to take part in the study was obtained from each participant before completing the questionnaire.

## Results

### Demographics

A total of 646 teachers took part in the study. The average age of participants was 44.54 years with a range of 24 to 76 years. There were 325 females and 321 males. 347 were from the government sector, and 299 were working in the private sector. As for educational qualification, 85 participants were graduates, 378 were post-graduates, 67 were doctorates and 116 gave their qualification as professional. Regarding level of teaching, 170 were teaching at College/Professional level, 196 were teaching in senior secondary level (up to 12 ^th^ standard or grade, equivalent to senior high school in USA), 139 were middle/junior school teachers (8 ^th^ standard or grade) and 141 were pre-primary or primary teachers (play school to 5 ^th^ standard). Full demographic data can be found under *Underlying data*. ^31^

### Statistical analysis

Cronbach alpha was calculated for all three scales to test their internal reliability. A level of more than 0.7 is considered adequate and a value above 0.8 is indicative of good reliability of the scale. The value for SSEIT was 0.832, for PSS 0.807, and for HBC the value was 0.866. Thus, all three scales showed good reliability.

Table 1 describes the correlation of emotional intelligence with age, stress and health behaviour and correlation of health behaviour and stress.

**Table 1.** Correlation analysis.

**EI and Age**	Pearson Correlation	-.010
	Sig. (2-tailed)	.804
	N	646
**EI and Stress**	Pearson Correlation	-.231
	Sig. (2-tailed)	.000
	N	646
**EI and Health Behaviour**	Pearson Correlation	.499
	Sig. (2-tailed)	.000
	N	646
**Stress and Health Behaviour**	Pearson Correlation	-.133
	Sig. (2-tailed)	.001
	N	646

^*^ P value <0.05 is significant.

This table clearly shows that there is no correlation between emotional intelligence and age. The coefficient r value of -.010 suggests a very weak and negative correlation between age and EI which is not statistically significant (p > 0.05). Between EI and Stress, a coefficient r value of -0.231 and p <0.01 means there is a negative correlation between EI and Stress which is statistically significant. Between EI and health behaviour the above table shows a coefficient r value of 0.499 and p value of <0.01, which suggests a statistically significant positive correlation between EI and health behaviour. Between stress and health behaviour the above table shows a coefficient r value -0.133 and a p value <0.01, which means a significant negative correlation between stress and health behaviour.

The results in Table 2 show that EI scores between male and female teachers were not significantly different. Similarly, there was no significant difference in EI between teachers of government institutes and teachers of private institutes.

**Table 2.** Effect of gender and type of institute on EI.

Levene's Test for Equality of Variances						t-test for Equality of Means				
		F	Sig.	t	Df	Sig. (2-tailed)	Mean Difference	Std. Error Difference	95% Confidence Interval of the Difference	
									Lower	Upper
EI and gender	Equal variances assumed	.891	.346		644	.528	-.60111	.95304	-2.47255	1.27033
	Equal variances not assumed				640.924	.529	-.60111	.95338	-2.47323	1.27101
EI of Government and private teachers	Equal variances assumed	.102	.749	.935	644	.350	.89292	.95531	-.98298	2.76882
	Equal variances not assumed			.935	630.488	.350	.89292	.95511	-.98266	2.76850

^*^ P value <0.05 is significant

Table 3 clearly suggests that there was no significant difference in EI scores among teachers with different educational qualifications and teachers at different level of teaching. In other words, educational qualification and level of teaching do not affect EI of teachers.

**Table 3.** Effect of educational qualification and level of teaching on EI.

EI and Educational Qualification								
	N	Mean	Std. Dev	Std. Error	95% Confidence Interval for Mean		Min	Max
					Lower Bound	Upper Bound		
Doctoral	67	128.6119	13.86729	1.69416	125.2294	131.9944	75.00	160.00
Grad	85	129.0588	12.63438	1.37039	126.3337	131.7840	97.00	159.00
Postgrad	378	128.2989	11.88754	.61143	127.0967	129.5012	68.00	158.00
Professional	116	127.0776	11.39119	1.05765	124.9826	129.1726	74.00	149.00
Total	646	128.2121	12.10560	.47629	127.2768	129.1473	68.00	160.00

ANOVA								
	Sum of Squares	df		Mean Square	F	Sig.		
Between Groups	223.808	3		74.603	.508	.677		
Within Groups	94298.138	642		146.882				
Total	94521.946	645						

EI and Level of Teaching								
Coll/Prof	170	127.8765	12.73081	.97641	125.9489	129.8040	75.00	160.00
Middle/Junior	139	127.7914	12.16819	1.03209	125.7506	129.8321	68.00	158.00
Pre/Primary	141	129.5816	11.12087	.93655	127.7300	131.4332	102.00	159.00
Senior/Sec	196	127.8163	12.20454	.87175	126.0971	129.5356	74.00	158.00
Total	646	128.2121	12.10560	.47629	127.2768	129.1473	68.00	160.00

ANOVA								
	Sum of Squares		df	Mean Square	F	Sig.		
Between Groups	338.890		3	112.963	.770	.511		
Within Groups	94183.055		642	146.703				
Total	94521.946		645					

^*^ P value <0.05 is significant

**Table 4.** Multiple Linear Regression Model.

Regression Statistics	
Multiple R	0.231599
R Square	0.053638
Adjusted R Square	0.050694
Standard Error	7.806544
Observations	646

The equation of fitted multiple linear regression model to show the behaviour of different score variables is: Y=47.4493−0.1446 X _1_ – 0.0101 X _2_

Where the dependent variable Y is representing the stress score variable and independent variables X _1_ and X _2_ are respectively EI and HBC score variables. One can estimate the value of Y based on given values of X _1_ and X _2._ The multiple R value is 0.2316 which is not very high but this model gives multiple regression coefficients -0.1446 and -0.0101 for EI and HBC respectively which are negative. The model shows the stress is negatively associated with EI and HBC.

To summarize the above results, emotional intelligence of teachers has no relation with age, gender, educational qualification, level of teaching or type of institute. It has a negative correlation with stress and a positive correlation with health behaviour. Further, health behaviour is inversely related to stress. Thus, teachers with low scores on EI and health behaviour are more likely to develop high stress and those with high EI and positive health behaviour are more likely to suffer less stress. The regression model shows that although the overall impact of EI and health behaviour on stress is not very large, nonetheless, both parameters independently affect stress and can be utilized as markers for future interventions.

## Discussion

Previous research has shown that EI has a positive correlation with stress and a negative correlation with health behaviour. Indian studies on teachers have shown conflicting results regarding the effect of demographic parameters like age and gender on EI and have shown some relation of EI with either educational qualification or level and type of teachers. No Indian study has assessed the health behaviour of teachers so far.

The results of the present study show that teachers’ EI is not affected by age, gender, educational qualification, level of teaching or type of institute. In ability measures, women consistently perform better than men, but, in self-report measures which measure trait EI, this is usually not observed. ^15^ In other words, women might be generally better in understanding and managing emotions, their own self-perception might not be very different from men. Our results are consistent with the studies showing similar trait EI levels among men and women teachers. The present study has adequate number of respondents from both genders making the results more reliable. While the ability model finds EI near to cognitive intelligence and thus increasing EI with age, experience, professional qualification seems justified, no such direct consequence can be drawn regarding trait EI. In fact, the notion that trait EI does not improve with age, experience, educational qualification, or career advancement is a valid reason for targeted intervention in improving EI and not presuming that it will get corrected over time. Whether it be the students or teachers, the focus on academic and professional qualification will improve their cognitive abilities and skills, but not their EI. This clearly is a vindication of the concept of trait EI, which presumes it to be a part of the personality and not related to cognitive abilities. Therefore, it follows that assessments and training of both cognitive and emotional aspects of individuals should be done in parallel, as focussing only on one aspect might not prepare one for the complexities and intricacies involved in the social and interpersonal relations.

The results also show that EI is not affected by educational qualification, level of teaching or type of institutes. Very few Indian studies have examined EI of teachers from different level of teaching or from different educational institutes and this precludes a generalization of their findings. A few studies have reported the association of EI with professional background or level of teaching but with limited sample size and without accounting for other confounding factors. ^13^ The present study is much wider in scope with representation of teachers right from pre-primary level up to higher professional institutes and colleges from both government and private sector. None of these attributes were significantly related to EI and this again corroborates the concept of trait EI as being independent of cognitive intelligence and acquired knowledge.

In line with accepted wisdom, EI had a significant negative correlation with stress, and this emphasizes the fact that teachers with low EI need to be properly counselled to prevent and manage stress so that they can function appropriately in the school. There was a positive correlation between EI and health behaviour which is similar to previous studies. Although health behaviour is a less studied subject and no Indian study has previously assessed health behaviour of teachers, it is an important parameter which evaluates the attitude towards a healthy lifestyle. Previous research has shown that heath behaviour of teachers can impact not only their own wellbeing but also that of students and some countries have studied the role of national or local programs targeting teachers for some specific health intervention like smoking cessation. Health behaviour has been found to be an effective coping strategy which can help in reducing stress. Our study also found a negative correlation between health behaviour and stress.

There are a few limitations of the study. First, all self-report measures have a potential for misrepresentation by participants. But this fact applies more in assessment of these parameters of individual participants, and very less when making correlation analysis between two parameters. Second, the pre-defined target of 100 subjects in each category was not reached for educational qualification, but this was a parameter which was only revealed later. The initial screening and sampling targeted teachers based on their gender and level of teaching which satisfied the desired numbers. Third, analysis between different dimensions of EI and health behaviour was not carried out, as the objectives of the study was primarily to determine the interrelation between the three main parameters and composite scores are more meaningful in that respect for planning any interventions for training purposes.

## Conclusion

To the best of our knowledge, this is the first Indian study which has evaluated emotional intelligence of teachers from different educational qualifications and teaching at different level and type of institutes using an internationally valid tool and assessing the impact of these factors on EI. This is also the first Indian study to examine health behavior of teachers. The study finds that trait emotional intelligence of teachers has a positive correlation with health behaviour and both trait EI and health behaviour independently affect stress. As the study involved teachers from different levels of teaching with different educational qualifications, the results are more generalized than previous research. The results of the study can influence certain practices at administrative level as well as clarifying certain debatable issues regarding the EI construct. Assessment of Emotional intelligence and health behaviour of teachers should be a part of their routine evaluation and training so that specific interventions to reduce stress and to improve their overall health and performance can be appropriately planned. This could be an important policy initiative for both public health and academics. Second, assigning teachers to administrative and other non-academic tasks can be helped by this data as selecting the best individual for a task needs an overall assessment of personality and not just academic credential or experience. Further, the results of the present study show that trait EI is an independent parameter which is not affected by age, gender, educational qualification and level or type of educational institute.

## Data Availability

Figshare: Data for study. https://doi.org/10.6084/m9.figshare.22262476.v2. ^31^ This project contains the following underlying data:
• Data file 1. Demographic and professional details • Data file 2. The three scales (questionnaires) and responses This project contains the following extended data:
• Questionnaire Data are available under the terms of the Creative Commons Zero “No rights reserved” data waiver (CC0 1.0 Public domain dedication).
