# Supplementary material for: Assessing trait emotional intelligence and its relationship with stress and health behaviour in the education sector: An empirical study from Uttarakhand, India
Source: F1000Res. 2023 Nov 8;12:320. Originally published 2023 Mar 23. [Version 4] doi: 10.12688/f1000research.131306.4 (PMC10432888; doi:10.12688/f1000research.131306.4)
Supplement: Peer review history

# Reviewer report: Reviewer response for version 2

**DOI:** 10.5256/f1000research.147919.r185345

**Published:** 2023-08-16

**Authors:** Tejpreet Kang

**Affiliations:** 1 Department of Human Development, Punjab Agricultural University, Ludhiana, Punjab, India

1. In the last line of the Introduction, it has been claimed that " *No such study has been conducted among teachers in India*". In the year 2016, my M.Sc student did a study on Occupational Stress in relation to EI among University teachers across different designations so a word "limited" or "few" will be better suited and appropriate. 
2. Use of the word "Type of Institute" in the whole text.
3. In hypothesis H1, instead of using "As a construct ___ teachers", you can use "There is no significant difference ___ teaching". 
4. H2 issue should be "There is no significant difference in stress based ___".
5. H3 could be "There is no significant difference in health behavior based _____". 
6. Year of publication of standard tools should be mentioned in the text. 
7. The tools should also be included in the references. 

Is the work clearly and accurately presented and does it cite the current literature?

Yes

If applicable, is the statistical analysis and its interpretation appropriate?

Yes

Are all the source data underlying the results available to ensure full reproducibility?

Yes

Is the study design appropriate and is the work technically sound?

Yes

Are the conclusions drawn adequately supported by the results?

Yes

Are sufficient details of methods and analysis provided to allow replication by others?

Yes

Reviewer Expertise:

Human Development and Family Studies

I confirm that I have read this submission and believe that I have an appropriate level of expertise to confirm that it is of an acceptable scientific standard.

# Response

**Published:** 2023-08-23

**Authors:** Mamta Pandey

**Affiliations:** Uttaranchal University, India

Response to second reviewer's comments:

 1. The last line in introduction specifically refers to studies on health behaviour. The study referred here evaluates EI and occupational stress, and we thank the reviewer for pointing this out and adding to our literature review. This paper had been added as a reference and cited in introduction in the section about stress. (Ref 20 in new version).

 2. Type of institute refers to government run or private sector.

 3. Significant added in first line in H1.

 4/5. H2 and H3 are not null hypothesis, but alternative hypotheses, which have been used directly for clarity and brevity.

 6. All the three standard scales used have been cited and referenced (Ref no 21, 29, and 30 in new version).

# Reviewer report: Reviewer response for version 2

**DOI:** 10.5256/f1000research.147919.r173799

**Published:** 2023-06-08

**Authors:** Shivani Agarwal

**Affiliations:** 1 KIET School of Management, KIET Group of Institutions, Ghaziabad, Uttar Pradesh, India

Approved.

Is the work clearly and accurately presented and does it cite the current literature?

Yes

If applicable, is the statistical analysis and its interpretation appropriate?

Yes

Are all the source data underlying the results available to ensure full reproducibility?

Yes

Is the study design appropriate and is the work technically sound?

Yes

Are the conclusions drawn adequately supported by the results?

Yes

Are sufficient details of methods and analysis provided to allow replication by others?

Yes

Reviewer Expertise:

Human Resource Management

I confirm that I have read this submission and believe that I have an appropriate level of expertise to confirm that it is of an acceptable scientific standard.

# Reviewer report: Reviewer response for version 1

**DOI:** 10.5256/f1000research.144136.r168321

**Published:** 2023-05-04

**Authors:** Shivani Agarwal

**Affiliations:** 1 KIET School of Management, KIET Group of Institutions, Ghaziabad, Uttar Pradesh, India

1. The introduction only presents the research context, in order to indicate the research motivation, the authors need to explain whether any paper has been or has not been published related to this topic to explain why the authors choose this topic for researching.
2. The research gap is not convincing, it needs to be re-interpreted.
3. Include additional content about the research methodology.
4. No research model and research hypothesis. These are very important for a study.
5. The reliability of the scale of the independent variables has not been tested yet.
6. As data belongs to Covid times, the authors can add Agarwal and Roshani (2023 ^1^), Agarwal and Jindal (2022 ^2^) and Mewafarosh and Agarwal (2021 ^3^).

Is the work clearly and accurately presented and does it cite the current literature?

Yes

If applicable, is the statistical analysis and its interpretation appropriate?

Yes

Are all the source data underlying the results available to ensure full reproducibility?

Yes

Is the study design appropriate and is the work technically sound?

Yes

Are the conclusions drawn adequately supported by the results?

Yes

Are sufficient details of methods and analysis provided to allow replication by others?

Yes

Reviewer Expertise:

Human Resource Management

I confirm that I have read this submission and believe that I have an appropriate level of expertise to confirm that it is of an acceptable scientific standard, however I have significant reservations, as outlined above.

## References

[1] : ‘Employee Experience during Covid-19 - Challenges and Opportunities’ in HR 4.0 Practices in the Post-COVID-19 Scenario. *AAP CRC* .2023; Reference source

[2] : Relieving Burnout through Job Design: A SEM Approach. *Ingeniería Solidaria* .2022;18(3) : 10.16925/2357-6014.2022.03.01 1-19 10.16925/2357-6014.2022.03.01

[3] : Influence of demographics variables on students subjective well‐being. *Journal of Public Affairs* .2022;22(S1) : 10.1002/pa.2749 10.1002/pa.2749

# Response

**Published:** 2023-05-06

**Authors:** Mamta Pandey

**Affiliations:** Uttaranchal University, India

Point 1 and 2.

 The review of literature mentions clearly that a lot of research has been done on emotional

 intelligence and stress, both in Indian and International context and some relevant papers

 have been cited. But the Indian studies on teachers' Emotional Intelligence have some

 limitations like small sample size, inclusion of only one type of teachers, use of indigenous

 scale which are not valid by international studies etc. Second, the impact of demographic factors like age and gender and professional factors like educational qualification and level of teaching is controversial which requires larger studies with an internationally valid tool to settle this issue. 

 Finally, Health Behaviour has been studied by a few international papers but no Indian study

 has evaluated health behaviour of teachers so far. This is an important parameter related to

 attitude towards a healthy life style which gained special attention during Covid Times. This

 was the motivation behind including this parameter in this study as well as inclusion of

 different levels of teachers. 

 Point 3. 

 Details about methodology have been already discussed. Initial data collection was convenient and online due to lockdown restrictions. Subsequently randomized purposive sampling was done by physical interviews and filling of questionnaires manually by participants. 

 Point 4

 During the literature search we had come across some papers which had used a structural

 equation model (SEM) to study relationship between EI and Other parameters but none of the

 studies have taken EI, Stress and Health Behaviour using a mediation model probably

 because EI and Health Behaviour are two distinct independent variables which independently

 affect stress but do not mediate the effect of each other. Based on the available literature we

 also formed a hypothesis suggesting the positive correlations between these two and a

 negative correlation with stress. The hypothesis and a schematic with research model have  been added as per instructions.

 Point 5

 All the three scales used in the study have been internationally used and tested in our studies

 also we tested the reliability of all the three scale was calculated using Cronbach alpha which

 has been mentioned in the result section.

 Point 6

 We have gone through the papers suggested which provide valuable inside regarding various

 parameters studied and statistical models but we are unable to include them in our literature

 review as the parameters used bear no direct relationship with our own parameters and have

 been done in different sample population. Also although our study were planned during covid

 times the full collection of data was done offline and covid itself was not a subject matter.
